# Multi-measure assessment of adherence to antiretroviral therapy among children under five years living with HIV in Jinja, Uganda

**DOI:** 10.1186/s12889-020-09430-w

**Published:** 2020-08-31

**Authors:** Jacquellyn Nambi Ssanyu, Mary Nakafeero, Fred Nuwaha

**Affiliations:** Makerere University School of Public Health, New Mulago Hospital Complex, P.O. Box 22864, Kampala, Uganda

**Keywords:** Adherence, Antiretroviral therapy, Children under five years, Uganda

## Abstract

**Background:**

Adherence to Antiretroviral Therapy (ART) is required to achieve HIV viral load suppression. However, children under 5 years in Jinja, Uganda, had been shown to have low HIV suppression rates. This study aimed to determine the level of ART non-adherence among these children and the associated factors.

**Methods:**

Data for the cross-sectional study was collected from April to July 2019, from caregivers of 206 children under 5 years living with HIV who were attending health facilities in Jinja and had been on ART for at least 3 months. Non-adherence was measured using a Visual Analog Scale that assessed both dosing and timing non-adherence, and by determining the Proportion of Days Covered by the medication. A questionnaire administered to the caregivers was used to collect the data, together with medical record review. A child was only considered adherent if they had adherence greater than 95% on all the measures. The data was analysed using Modified Poisson Regression, taking a *p*-value less than 0.05 as statistically significant.

**Results:**

Of the 206 children, 73.8% were older than 2 years, and 52.9% were female. Likewise, the majority of caregivers were female (93.7%). Using the combined adherence measure, 57.3% of the children were categorised as non-adherent. School/day-care attendance, Prevalence Ratio (PR) = 1.25 (*p* = 0.042), the caregiver having higher than a primary school education, PR = 0.72 (*p* = 0.044) and satisfaction with the quality of service at the health facility, PR = 0.97 (*p* < 0.001) were associated with non-adherence. Household food insecurity was also associated with non-adherence: PR = 1.55 (*p* = 0.011) for mild food insecurity, PR = 1.75 (*p* = 0.001) for moderate insecurity and PR = 1.48 (*p* = 0.015) for severe food insecurity.

**Conclusions:**

Children under 5 years in Jinja had a high level of ART non-adherence. It is important to engage schools to support adherence among children living with HIV. Addressing household food insecurity and improving the quality of paediatric ART services would also reduce the barriers to optimal adherence.

## Background

Since its introduction, Antiretroviral Therapy (ART) has gradually led to a reduction in the number of HIV related deaths globally [1], and the World Health Organization (WHO) recommends treatment for all people diagnosed with HIV regardless of the clinical stage [2]. In Uganda, children diagnosed with HIV start ART at the earliest opportunity, regardless of clinical stage, to suppress the viral load to undetectable levels [3]. Perfect or near-perfect adherence to ART, defined as adherence over 95%, is required to achieve full viral load suppression [4]. However, viral load suppression is lowest among children under 15 years in Uganda at 39.3% compared with 59.6% among those 15 years and older [5]. In Jinja district, specifically, HIV suppression rates recorded among children under 5 years between January and October 2018 were below the national target of 90% at 58.0%, and lower than the 71.8% recorded for children 5 to 14 years old and the 89.7% for patients 15 years and older [6].

A study done in Western Uganda assessing paediatric ART adherence found that children older than 10 years were more likely to adhere to ART than the younger ones [7]. Factors like caregiver’s knowledge of their HIV status and differences between health facilities were found to influence adherence [7]. Haberer, Kiwanuka [8] also found hospitalization of the child, liquid formulation use and caregiver alcohol use to be associated with less than 90% adherence among children aged 2 to 10 years in Western Uganda. However, most research on paediatric ART adherence in Uganda and elsewhere has mostly been among adolescents [9, 10]. For studies that include children under five, these children have been grouped with the older ones [7, 8]. This has the potential of disguising adherence challenges specific to children under five. Moreover, self-reports were the prevalent measure of non-adherence used. Yet, given the limitations of the different adherence measures, WHO recommends a multi-method approach to measuring ART non-adherence [11].

This study, therefore, aimed to determine the level of ART non-adherence among children under the age of five in Jinja district using an objective and a subjective measure of adherence, to increase the likelihood of identifying non-adherence. The study also intended to determine the child, caregiver, drug regimen and health system factors that influence this non-adherence. The study’s findings are expected to contribute to understanding barriers to optimal ART adherence among these children and guide the design of interventions to address them.

## Methods

### Study setting

Data was collected from April to July 2019, in health facilities that offer paediatric ART services in Jinja. Jinja is a district in the south-eastern part of Uganda, with a population of about 471,242 people [12]. At the district level, the public health system in Uganda is decentralized, with Village Health Teams making up the lowest level, followed by Health Centre II facilities (HCII’s), HCIII’s, HCIV’s and the district and regional hospitals. Data was collected from Jinja Regional Referral Hospital, 2 Non-Government Organization-run health facilities, the 5 HCIV’s and 9 HCIII’s. These health facilities all provide paediatric ART services at no charge.

### Study design

A cross-sectional study design was used. Quantitative data was collected through a health facility-based survey and medical record review.

### Participants

The study population was children under 5 years living with HIV who were receiving ART services from health facilities in Jinja, and had been on ART for at least 3 months, as recorded in their medical records. The respondents were the caregivers with whom the children presented to the health facilities on the days of data collection. Only children who had lived with their caregiver for at least 1 month were included in the study. These were sampled consecutively as they were presented for care and, for caregivers with more than one eligible child, the first child to be presented was included. Eligible children whose caregivers declined to give consent to participate were excluded from the study.

### Data collection, variables and measurement

An interviewer-administered electronic questionnaire developed by the authors was used to collect data from eligible caregivers using KoBo Toolbox kit [13] installed on mobile phones. The questionnaire was provided in English and also translated into Lusoga, the language predominantly spoken in Jinja. An English version of the questionnaire is provided in Additional file 1. Data was extracted from medical records of children whose caregivers had been interviewed using an electronic data abstraction form designed for this study. This form is available in Additional file 2. The study tools had been pretested on five children in a health facility in a neighbouring district. Data was collected by a male and female research assistant, who had training in Nursing and Social Sciences, respectively. Both had experience in data collection and were fluent in both Lusoga and English. They were also trained in the study procedures and ethical conduct of research before data collection commenced.

The outcome variable, non-adherence to ART, was measured using a Visual Analogue Scale (VAS) and the Proportion of Days Covered (PDC). The VAS was based on the modified Medication Adherence Self-Report Inventory questionnaire, which includes two VAS items: one part measures how much of the prescribed medicine is taken (VAS-dose) while the second part measures how much of it is taken within 2 hours of the recommended time (VAS-timing), both measured over 30 days [14]. Through medical record review, concurrent adherence to all medicines in the ART regimen for each child was also determined by calculating the PDC, the proportion of days over 90 days when the child had a supply of all drugs in their ART regimen. Adherence was categorised at the 95% cut-off on the VAS-dose, VAS-timing and the PDC measures, and only children with adherence levels of 95% or higher on all three measures were considered adherent.

Data was also collected on the exposure variables: the child, drug regimen, health system and caregiver factors. The child factors were age, gender, place of delivery, school/day-care attendance, illness in the past 2 weeks and clinical stage on ART initiation, which were measured using the questionnaire. The child’s most recent viral load reading before the date of data collection was recorded from their medical records. Data on drug regimen factors, including the drug combination the child was on, the experience of side effects and drug regimen changes since ART initiation was obtained from medical records. Health system factors were measured using the questionnaire. These included: the type of health facility, the physician-patient relationship which was measured using 13 items from the Patient Communication Behaviours Scale [15] and the caregiver satisfaction with the quality of service at the child’s ART clinic, assessed using a 9-item scale adopted from Ivy, Ng [16].

The caregiver factors were assessed through the questionnaire, and they included: age, sex, highest education level attained, marital status, place of residence, HIV status, relation to the child, the main source of financial support, beliefs about medicine, alcohol use and household food security. The caregiver’s beliefs about medicine were assessed using 10 questions from the 18-item Beliefs About Medicine questionnaire (BMQ) [17]. These included 5 questions assessing beliefs about necessity of the medicine (BMQ-Necessity scale), and 5 questions assessing concerns about the medicine’s negative effects (BMQ-Concern scale). Statements in the BMQ were scored on a Likert-type scale that ranged from 1, for strongly disagree to 5, for strongly agree. The maximum score on both the BMQ-Necessity and BMQ- Concern scales was 25, and using a cut-off of 12.5, caregiver beliefs about medicine could be classified as “Accepting”, “Ambivalent”, “Indifferent” or “Skeptical” [18, 19]. Household food security was measured using the Household Food Insecurity Access Scale (HFIAS), which categorised households as “Food secure”, “Mildly food insecure”, “Moderately food insecure” or “Severely food insecure” [20]. Caregiver alcohol use was assessed over a period of 30 days before the date of data collection using the question, “How often have you had an alcoholic drink in the last 30 days?”, with responses, “Never”, “Once a month”, “2 or 3 times a month”, “Once or twice a week”, “3 or 4 times a week”, “Nearly every day” and “Daily”.

### Study size

The formula for sample size determination of cross-sectional studies [21] was used to estimate the number of children to sample. The prevalence of ART non-adherence was estimated at 21% [7] and, using a precision of 5%, the sample size was calculated and adjusted for a finite population of 305, the number of children active on ART in Jinja as of September 2018. The final sample size was then adjusted for non-response of 20% to give a minimum sample size of 168 children.

### Statistical analysis

Data was analysed using STATA version 14 (StataCorp LP, TX, USA). Means with standard deviations were used to summarize normally distributed continuous variables, while medians with interquartile ranges were used for continuous variables that were non-normally distributed. Frequencies and their corresponding percentages were used for the categorical variables. The outcome variable, non-adherence measured by the combined VAS and PDC measures, was presented as a proportion.

Modified Poisson Regression was used for bivariate and multivariable analysis while associations were measured using Prevalence Ratios (PRs). Exposure variables with *p*-values less than 0.1 at bivariate analysis were considered for inclusion into the multivariable analysis model. Multi-collinearity between the candidate variables was examined and a correlation coefficient greater than 0.4 was considered high collinearity. Stepwise elimination was used to build the multivariable model, where elimination and addition of variables were based on the Akaike Information Criteria and variables specified as important from previous literature. A *p*-value of less than 0.05 was considered statistically significant for all analyses.

## Results

### Characteristics of the study participants

A total of 206 children under 5 years were included in the study. The majority of the children (73.8%) were older than 2 years, 109 (52.9%) of them were female, and 72 (34.9%) were in school/day-care. Of all the children included, 34.9% had their most recent viral load recorded as less than 1000 copies/ml, while 37.4% did not have a record for the latest viral test result.

Out of the 206 caregivers interviewed, 48.1% were aged from 25 to 34 years, and 193 (93.7%) were female. Only 50 (24.3%) of the caregivers were unemployed. The majority (73.8%) were the biological parents of the children they had presented for care. Most were living with HIV (79.1%), and of these 98.8% were on ART. (Table 1).

**Table 1:** Child and caregiver descriptive characteristics (n = 206)

Characteristic	Frequency (%)
***Children***	
**Age (months)**	
6 to 24	54 (26.2)
25 to 59	152 (73.8)
**Health facility type**	
Public health facility	142 (68.9)
NGO health facility	64 (31.1)
**Current ART regimen**	
ABC + 3TC + LPV/r	124 (60.2)
Other ABC-based regimen^a^	50 (24.3)
Other AZT-based regimen^b^	32 (15.5)
**Duration on ART (months)**	
Median (IQR)	18.5 (20.7)
**Most recent viral load**	
< 1000 copies/ml	72 (34.9)
≥ 1000 copies/ml	57 (27.7))
Missing	77 (37.4)
***Caregivers***	
**Age (years)**	
18 to 24	41 (19.9)
25 to 34	99 (48.1)
35 to 44	44 (21.3)
45 to 70	22 (10.7)
**Highest education level attained**	
None	19 (9.2)
Primary school^c^	90 (43.7)
Higher^d^	97 (47.1)
**Marital status**	
Never married	28 (13.6)
Living with partner but not married	124 (60.2)
Married	10 (4.8)
Divorced/Separated	35 (17.0)
Widow/Widower	9 (4.4)
**Residence**	
Rural	128 (62.1)
Urban	78 (37.9)
**Monthly income (Uganda shillings)**	
< 130,000	93 (67.4)
≥ 130,000	45 (32.6)
**HIV Status**	
Positive	163 (79.1)
Negative	40 (19.4)
Didn’t Know	3 (1.5)
**Living with HIV and on ART (*n*** **= 163)**	
Yes	161 (98.8)
No	2 (1.2)
**Relation to the child**	
Biological parent	152 (73.8)
Other blood relative	17 (8.2)
Non-blood relation	37 (18.0)
**Alcohol use in the last 30 days**	
Never	192 (93.2)
Ever used	14 (6.8)

^a^ ABC + 3TC + EFV, ABC + 3TC + NVP

^b^ AZT + 3TC + LPV/r, AZT + 3TC + NVP, AZT + 3TC + EFV

^c^ Primary school is equivalent to about 7 years of formal education

^d^ Higher: Secondary school (6 years) or tertiary education

### Level of non-adherence to ART

Using the 30-day VAS scale, 38.4% of the caregivers reported having given the children less than 95% of their doses (VAS-dose) while 53.4% of them had not administered their children’s medicine within 2 h of the correct time (VAS-timing). Using the PDC measure, 16.5% of the children were categorised as non-adherent to their ART medication in the past 90 days. Applying the combined VAS-dose, VAS-timing and PDC measure, 118 (57.3%) of the children were found to be non-adherent.

On the other hand, healthcare providers in Uganda also routinely assess and record adherence at every child’s clinic visit. From record review, only 5.8% of all the children included in this study had been categorised by their healthcare providers at their most recent visit as having an adherence level below 95%.

### Factors associated with non-adherence

At bivariate analysis, only school/day-care attendance was significantly associated with non-adherence among the child factors. (Table 2) Caregiver factors significantly associated with non-adherence at bivariate analysis were caregiver HIV status, their beliefs about medicine and household food security. Among the health system factors, type of health facility and caregiver satisfaction with the quality of care at the health facility were significantly associated with non-adherence at bivariate analysis. None of the drug regimen factors was significantly associated with non-adherence at this level.

**Table 2:** Factors associated with non-adherence at bivariate analysis

	Adherent	Non-adherent					
	(*n* = 88)	(*n* = 118)					
Factor	n (%)	n (%)	Unadjusted PR		Confidence Interval		*p*-value
***Child factors***							
**Age (months)**							
6 to 24	24 (44.4)	30 (55.6)	1.00				
25 to 59	64 (42.1)	88 (57.9)	1.04		(0.79–1.37)		0.769
**Sex**							
Male	35 (36.1)	62 (63.9)	1.00				
Female	53 (48.6)	56 (51.4)	0.80		(0.63–1.02)		0.070
**Place of delivery**							
Home	9 (34.6)	17 (65.4)	1.00				
Public Health Facility	56 (50.5)	55 (49.5)	0.76		(0.54–1.06)		0.107
Private Health Facility	17 (36.2)	30 (63.8)	0.98		(0.69–1.39)		0.894
Didn’t know	6 (27.3)	16 (72.7)	1.11		(0.76–1.63)		0.583
**School/Day care attendance**							
No	65 (48.5)	69 (51.5)	1.00				
Yes	23 (31.9)	49 (68.1)	1.32		(1.05–1.67)		0.017
**Illness in the past two weeks**							
No	65 (45.5)	78 (54.5)	1.00				
Yes	23 (36.5)	40 (63.5)	1.16		(0.92–1.48)		0.215
**Clinical stage on ART initiation**							
I	40 (42.6)	54 (57.4)	1.00				
II	18 (47.4)	20 (52.6)	0.92		(0.65–1.30)		0.623
III	8 (32.0)	17 (68.0)	1.18		(0.86–1.63)		0.304
IV	2 (25.0)	6 (75.0)	1.31		(0.84–2.02)		0.232
***Caregiver factors***							
**Age (years)**							
18 to 24	21 (51.2)	20 (48.8)	1.00				
25 to 34	40 (40.4)	59 (59.6)	1.22		(0.86–1.74)		0.267
35 to 44	17 (38.6)	27 (61.4)	1.26		(0.85–1.86)		0.252
45 to 70	10 (45.5)	12 (54.5)	1.12		(0.68–1.83)		0.658
**Sex**							
Male	4 (30.8)	9 (69.2)	1.00				
Female	84 (43.5)	109 (56.5)	0.82		(0.56–1.20)		0.299
**Highest education level attained**							
None	7 (36.8)	12 (63.2)	1.00				
Primary school	36 (40.0)	54 (60.0)	0.95		(0.65–1.39)		0.793
Higher	45 (46.4)	52 (53.6)	0.85		(0.57–1.26)		0.411
**Marital status**							
Never married	15 (53.6)	13 (46.4)	1.00				
Living with partner but unmarried	54 (43.6)	70 (56.4)	1.22		(0.79–1.86)		0.371
Married	3 (30.0)	7 (70.0)	1.51		(0.85–2.67)		0.158
Divorced/Separated	12 (34.3)	23 (65.7)	1.42		(0.89–2.25)		0.143
Widow/Widower	4 (44.4)	5 (55.6)	1.20		(0.59–2.43)		0.620
**Residence**							
Rural	52 (40.6)	76 (59.4)	1.00				
Urban	36 (46.2)	42 (53.8)	0.91		(0.71–1.17)		0.446
**HIV Status**							
Negative	11 (27.5)	29 (72.5)	1.00				
Positive	75 (46.0)	88 (54.0)	0.74		(0.59–0.95)		0.015
Didn’t Know	2 (66.7)	1 (33.3)	0.46		(0.09–2.31)		0.346
**Relation to the child**							
Biological parent	69 (45.4)	83 (54.6)	1.00				
Other blood relative	7 (41.2)	10 (58.8)	1.08		(0.70–1.65)		0.731
Non-blood relation	12 (32.4)	25 (67.6)	1.24		(0.95–1.62)		0.118
**Main source of financial support**							
From income	20 (33.3)	40 (66.7)	1.00				
From family	22 (48.9)	23 (51.1)	0.77		(0.55–1.07)		0.123
From partner	42 (45.2)	51 (54.8)	0.82		(0.64–1.06)		0.137
From friends and charities	4 (50.0)	4 (50.0)	0.75		(0.37–1.54)		0.432
**Beliefs about medicine**							
Ambivalent	71 (49.0)	74 (51.0)	1.00				
Accepting or indifferent	17 (27.9)	44 (72.1)	1.41		(1.13–1.77)		0.002
**Alcohol use in the last 30 days**							
Never	84 (43.8)	108 (56.3)	1.00				
Ever used	4 (28.6)	10 (71.4)	1.27		(0.89–1.81)		0.187
**Household food security**							
Food secure	61 (59.8)	41 (40.2)	1.00				
Mild insecurity	8 (26.7)	22 (73.3)	1.82		(1.32–2.51)		< 0.001
Moderate insecurity	7 (26.9)	19 (73.1)	1.82		(1.30–2.54)		< 0.001
Severe insecurity	12 (25.0)	36 (75.0)	1.87		(1.40–2.49)		< 0.001
***Drug regimen characteristics***							
**ART regimen currently on**							
ABC + 3TC + LPV/r	50 (40.3)	74 (59.7)	1.00				
Other ABC-based regimen	26 (52.0)	24 (48.0)	0.80		(0.58–1.11)		0.187
Other AZT-based regimen	12 (37.5)	20 (62.5)	1.05		(0.77–1.42)		0.767
**ART regimen change**							
No	47 (46.1)	55 (53.9)	1.00				
Yes	41 (39.4)	63 (60.6)	1.12		(0.89–1.42)		0.337
**Side effects reported**							
No	79 (41.4)	112 (58.6)	1.00				
Yes	9 (60.0)	6 (40.0)	0.68		(0.36–1.28)		0.236
***Health system factors***							
**Health facility**							
Public health facility	41 (28.9)	101 (71.1)	1.00				
NGO health facility	47 (73.4)	17 (26.6)	0.37		(0.24–0.57)		< 0.001
**Physician-patient relationship**							
Mean score (S.D)	62.2 (7.4)	61.1 (6.4)	0.99		(0.98–1.01)		0.264
**Satisfaction**							
Mean score (S.D)	83.5 (7.1)	78.9 (7.0)	0.97		(0.96–0.98)		< 0.001

Six variables were included in the final multivariable analysis model; sex of the child, school/day-care attendance, the caregiver’s highest education level attained, beliefs about medicine, household food security and caregiver satisfaction with the quality of service at the health facility. (Table 3). After controlling for other variables, among the child factors, only school/day-care attendance was significantly associated with non-adherence: children who were in school/day-care were 1.25 times as likely to be non-adherent as compared to those who were not in school/day-care (*p* = 0.042; 95% C.I.: 1.01, 1.55).

**Table 3:** Factors associated with non-adherence at multivariable analysis

	Unadjusted PR	Confidence interval	*p*-value	Adjusted PR	Confidence interval	*p*-value
Factor						
***Child factors***						
**Sex**						
Male	1.00			1.00		
Female	0.80	(0.63–1.02)	0.070	0.86	(0.68–1.08)	0.193
**School/Day-care attendance**						
No	1.00			1.00		
Yes	1.32	(1.05–1.67)	0.017	1.25	(1.01–1.55)	**0.042**
***Caregiver factors***						
**Highest education level attained**						
None	1.00			1.00		
Primary school	0.95	(0.65–1.39)	0.793	0.82	(0.60–1.13)	0.232
Higher	0.85	(0.57–1.26)	0.411	0.72	(0.52–0.99)	**0.044**
**Beliefs about medicine**						
Ambivalent	1.00			1.00		
Accepting or indifferent	1.41	(1.13–1.77)	0.002	1.16	(0.92–1.47)	0.214
** Household food security**						
Food secure	1.00			1.00		
Mild insecurity	1.82	(1.32–2.51)	< 0.001	1.55	(1.10–2.16)	**0.011**
Moderate insecurity	1.82	(1.30–2.54)	< 0.001	1.75	(1.25–2.45)	**0.001**
Severe insecurity	1.87	(1.40–2.49)	< 0.001	1.48	(1.08–2.02)	**0.015**
***Health system factors***						
**Satisfaction with the quality of care**						
Mean score	0.97	(0.96–0.98)	< 0.001	0.97	(0.96–0.99)	**< 0.001**

Caregivers who had higher than a primary school education were 0.72 times as likely to have a non-adherent child as compared to those with no education at all (*p* = 0.044, 95% C.I.: 0.52, 0.99). There was no significant difference between those who had only attained a primary school education and those who had no education. Non-adherence was also found to be more prevalent in households with food insecurity. The prevalence increased with increasing food insecurity from a PR of 1.55 (*p* = 0.011, 95% C.I.: 1.10, 2.16) for those with mild food insecurity; to a PR of 1.75 (*p* = 0.001, 95% C.I.: 1.25, 2.45) for moderate food insecurity. However, households with severe food insecurity had a lower prevalence of non-adherence, PR = 1.48 (*p* = 0.015, 95% C.I.: 1.08, 2.02), compared to those with mild and moderate food insecurity.

Satisfaction with the quality of services at the child’s ART clinic was the only health system factor significantly associated with non-adherence. The more satisfied a caregiver was with the quality of services at the health facility their child attended, the less likely their child was to be non-adherent to their ART medication, PR = 0.97 (*p*-value < 0.001; 95% C.I.: 0.96, 0.99).

## Discussion

This study aimed to determine the level of non-adherence to ART among children under 5 years in Jinja district Uganda, and the factors associated with it. Inasmuch as optimal adherence is important for achieving viral load suppression and delaying the emergence of drug resistance, findings from the study showed children under 5 years in Jinja to have a high level of non-adherence to ART. Non-adherence was more prevalent among children who attended school/day-care and those from households with food insecurity. It was less prevalent among children with caregivers who had higher than a primary school education and those with increased satisfaction with the quality of service received at the child’s ART clinic.

Most previous paediatric ART adherence research has studied children under 5 years combined with children of older age groups, using cut-offs varying from 80 to 95%. Most of these studies have also reported lower levels of non-adherence than what was found in this study. Wadunde, Tuhebwe [7] studied children aged 0 to 14 years in Western Uganda and found the level of non-adherence to be 21% using self-reports at a 90% cut off. This is similar to the 30% found among 2 to 19-year-old children in Tanzania at an 80% cut-off using pill counts [22], and the 9.7% among children under 15 years in Ethiopia using self-reports at a 95% cut-off [23]. Davies, Boulle [24] also reported a lower non-adherence level among children under 5 years in South Africa using medicine return at a 90% cut-off. However, studies that utilised combined measures of adherence were able to identify more non-adherent children and found similarly high levels of non-adherence as was found in our study. Nsheha, Dow [25] found non-adherence among children aged 2 to 17 years in Tanzania to be as high as 75.4% when they were subject to three measures of adherence: a two-day self-report, a one-month VAS and unannounced pill counts.

Differences were observed between the adherence measures used. More children were categorised as non-adherent by VAS than PDC, suggesting that many caregivers collect the ART medicines for their children from the clinics but fail to administer them at home. Providing ART at no cost in ART clinics in Uganda eliminates the biggest financial barrier to access, even if other barriers at home still prevail. It is also notable that healthcare providers during routine practice were only able to identify a few of the non-adherent children. This implies that relying on appointment keeping at the clinics alone to measure non-adherence, as is the practice in Uganda, may not be sufficient to identify and support non-adherent children.

Children who were in school/day-care were found to be more likely to be non-adherent and this could be attributed to the differences in daily schedules between these children and their caregivers, which make it easy to skip doses. Moreover, even if the caregiver remembers to administer the drugs after the scheduled dosing time, there would be no way of quickly administering the medication if the child has already left for school/day-care. The stigma faced by children living with HIV and their caregivers also makes it hard for the caregivers to entrust school teachers with administering the medicine at school. Nyogea, Mtenga [22] found unfavourable school environments to be a barrier to optimal adherence among children on ART in Tanzania.

Food insecurity was found to be associated with non-adherence and this would be expected as caregivers who are afraid of the effects of administering food on an empty stomach skip their children’s doses as they wait for food to be available. This finding is also similar to what Ndayikeje, Wilson [26] found in Rwanda where lack of food to take with tablets was associated with non-adherence to ART among children aged 1 to 18 years. Likewise, parents and guardians of children living with HIV in Western Kenya perceived lack of food as a barrier to their children’s optimum adherence to antiretroviral therapy. They feared the effects of administering the medicine without food because they thought the drugs required administration with food as they were too strong [27].

Caregivers who had higher than a primary school education were less likely to have non-adherent children than those with no education at all. Education increases the ability of caregivers to read and understand dosing instructions, and would be expected to improve adherence. This finding is similar to what Davies, Boulle [24] found among young children in South Africa, where secondary education of the caregivers was positively associated with adherence to antiretroviral therapy among the children.

As would be expected, children of caregivers who were satisfied with the quality of service at the health facilities were less likely to be non-adherent. The scale used to measure satisfaction with the quality of service at the health facility measured attributes such as the physical environment, pharmacy service, waiting time and the way the healthcare provider related with the children and caregivers [16]. Good quality services motivate caregivers to keep appointments to collect medicine. Nabukeera-Barungi, Elyanu [9] also found supportive health care workers and short waiting time at the health facility to be facilitators of adherence among adolescents in Uganda. It is, however, surprising that none of the drug regimen characteristics was significantly associated with non-adherence as had been reported in previous studies [22, 24, 26].

### Study limitations

A VAS is a subjective measure and is prone to reporting bias which may have underestimated non-adherence, especially as the data was obtained from caregivers. Drug refill data also assumes that all collected medicine is administered as instructed, and this may have also underestimated non-adherence. On the other hand, drug refill data may fail to reveal previous excess refills which may assume non-adherence where there is none, and thus overestimate non-adherence. Nonetheless, using the two measures combined in this study increased the ability to identify non-adherence. Direct objective measures of adherence such as therapeutic drug monitoring and medication event monitoring systems, where they can be used, are recommended for a more accurate assessment of adherence.

## Conclusions

Findings from this study revealed that children under 5 years in Jinja district had a high level of non-adherence to ART. School attendance and food insecurity were found to be associated with increased non-adherence. On the other hand, increased satisfaction with the quality of services at health facilities and the caregiver having higher than a primary school education were associated with reduced non-adherence to ART among these children.

It is therefore important to engage schools and day-care centres to support adherence to ART among young children living with HIV and to ensure continuous adherence to therapy even when the child is at school/day-care. Caregivers should also be empowered economically to guarantee food security. These should also be educated on less costly child feeding practices to ensure that children are tended to even in times of food scarcity. At the health facilities, constant review and improvement of paediatric ART clinic services should be prioritised to further minimise the barriers to adherence within the health system.

## Supplementary information

**Additional file 1.** English version of the questionnaire: English language version of the questionnaire for interviewing caregivers.

**Additional file 2.** Data abstraction form: Data abstraction form used for extraction of the children’s medical records.

## Data Availability

Data analysed during this study can be made available to all interested researchers upon reasonable request directed to the corresponding author, Ms. Jacquellyn Nambi Ssanyu (sanyukajacque@gmail.com).

## Abbreviations

3TC: Lamivudine
ABC: Abacavir
AIDS: Acquired Immune Deficiency Syndrome
ART: Antiretroviral therapy
EFV: Efavirenz
HFIAS: Household Food Insecurity Access Scale
HIV: Human Immunodeficiency Virus
LPV/r: Ritonavir-boosted Lopinavir
NVP: Nevirapine
PDC: Proportion of Days Covered
VAS: Visual Analog Scale
WHO: World Health Organization.
