# Supplementary material for: Multi-measure assessment of adherence to antiretroviral therapy among children under five years living with HIV in Jinja, Uganda
Source: BMC Public Health. 2020 Aug 31;20:1319. doi: 10.1186/s12889-020-09430-w (PMC7457490; doi:10.1186/s12889-020-09430-w)
Supplement: Supplementary file 1 — Additional file 1. English version of the questionnaire: English language version of the questionnaire for interviewing caregivers. [file 12889_2020_9430_MOESM1_ESM.pdf]

QUESTIONNAIRE:

Factors associated with non-adherence to anti-retroviral therapy among HIV positive children under five years in Jinja district.

Hello. My name is ____________ and I am a ______________ from ______________. I am going to ask you a few questions about your child and their experience with their anti-retroviral therapy. I will also ask you some questions about you and your family and your experience with this health facility to understand the factors that affect adherence to anti-retroviral therapy among children less than five years old. This questionnaire takes approximately 30-45 minutes. It is completely voluntary, and you can choose to skip a question or end the survey at any time. All information will be kept confidential and your name will not be attached to any of the information. Do you have any questions before we begin?

**Code**		**Code**	
date_int	Date of interview: (dd/mm/yyyy)	intr_id	Interviewer number:
stime	Start time of interview: (am/pm)	part_id	Participant ID:
etime	End time of interview: (am/pm)	facility	Health facility:

SCREENING QUESTIONS

**No.**	**Code**	**Question**	**Responses**
1.	age_cd	How old is this child?	______________ (age in months)
2.	dur_art	How long has this child been on ART?	______________ (time in months)
3.	timelength	How long have you lived with this child?	______________ (months)

SECTION A: Child characteristics

First, I am going to ask you some questions about your child

**No.**	**Code**	**Question**	**Responses**
4.	sex_cd	Sex?	1. Male [0]<br>2. Female [1]
5.	pod	Where was this child delivered?	1. Home [0]<br>2. Public Health Facility [1]<br>3. Private Health Facility [2]<br>4. Don’t know [99]
6.	school	Is this child in school/daycare?	1. Yes [1]<br>2. No [0]
7. (a)	vacc_card	Do you have your child’s vaccination card with you?	1. Yes [1]<br>2. No (Skip the next question) [0]
7 (b)	vacc_stat	Verify with the vaccination card if the child has received all the vaccines they are scheduled to have received by this age?	1. Yes [1]<br>2. No [0]

8.	birth_order	What is this child's birth order?	(number)
9.	sib_art	Is any of this child's siblings (biological and non-biological) on ART?	1. Yes [1]<br>2. No [0]<br>3. N/A (Has no siblings) [999]
10.	side_eff	Has your child experienced any side effects with their HIV medication?	1. Yes [1]<br>2. No [0]<br>3. Don't know [99]
11.	ill	Has the child been ill at any time in the last one month?	1. Yes [1]<br>2. No [0]<br>3. Don't know [99]

SECTION B: Caregiver characteristics

Now I am going to ask you some questions about you and your health

**No.**	**Code**	**Question**	**Responses**
12.	age_cg	How old are you?	(age in complete years)
13.	sex_cg	Sex	1. Male [0]<br>2. Female [1]
14.	tribe	What is your tribe?	
15.	residence	Do you live in an urban or rural area?	1. Urban [1]<br>2. Rural [0]
16.	religion	What is your religion?	1. Protestant [1]<br>2. Catholic [2]<br>3. Moslem [3]<br>4. Seventh Day Adventist [4]<br>5. Saved/Pentecostal [5]<br>6. None [0]<br>7. Other (specify) [6] religion_b
17.	educ	What is the highest education level you have attained?	1. None [0]<br>2. Primary [1]<br>3. O level [2]<br>4. A level [3]<br>5. Vocational [4]<br>6. Diploma [5]<br>7. Degree [6]
18.	maristat	What is your marital status?	1. Never married [0]<br>2. Living with partner but not married [1]<br>3. Married [2]<br>4. Divorced/Separated [3]<br>5. Widow/Widower [4]
19.	occup	What are you currently doing for a living?	1. No job/ jobless [0]<br>2. Farmer [1]<br>3. Casual labour [2]<br>4. Housewife/Househusband [3]<br>5. Small trader [4]<br>6. Police/military [5]<br>7. Office/clerical [6]<br>8. Professional (including health workers and teachers) [7]<br>9. Service (Restaurant, Hotel, etc) [8]<br>10. Student [9]

			11. Other (specify) [10] occup_b
20.	income	Approximately, how much do you earn per month? (Gross pay)	(Uganda shillings)
21.	support	What is your main source of financial support presently? *(select one response)*	1. From your income [0]<br>2. Support from family [1]<br>3. Support from partner [2]<br>4. Support from friends [3]<br>5. Social support (from charities, churches, organizations, etc) [4]
22.	hiv	What is your HIV status?	1. Positive [1]<br>2. Negative (Skip Q12) [0]<br>3. Don't know (Skip Q12) [99]
23.	art	If HIV positive, are you on ART?	1. Yes [1]<br>2. No [0]
24.	means	What means of transport do you most often use to get to this clinic to collect medicine for the child?	1. Walking [0]<br>2. Driving (private car or special hire) [1]<br>3. Boda boda [2]<br>4. Taxi [3]<br>5. Bicycle [4]<br>6. Other (Specify) [5] means_b
25.	fare	How much do you typically spend on transport to the clinic for you and the child to collect medicine for the child (to and fro fare)?	(Uganda shillings)
26.	fare_trb	Do you ever have trouble finding money for transport for you and the child to the clinic?	1. Rarely [0]<br>2. Sometimes [1]<br>3. Often [2]
27.	time	How long does it typically take you to get from your home to the clinic to collect medicine for the child (one-way)?	(minutes)
28.	chn_no	How many children do you take care of (biological and non-biological)?	(number)
29.	chn_live	How many of these children live with you?	…………(number)
30.	relation	What is your relation to this child?	1. Biological parent [0]<br>2. Non-biological legal guardian [1]<br>3. Other blood relative [2]<br>4. Other non-blood relationship [3]

Beliefs about medicine

I would like to ask you about your personal views about medicines prescribed for your child. These are statements other people have made about their medication. Please indicate the extent to which you agree or disagree with them by selecting whether you strongly agree, agree, are uncertain, disagree or strongly disagree. There are no right or wrong answers. I am interested in your personal views.

31.	bmqs1	My child's health presently depends on their antiretroviral (ARV) medicines	1. Strongly agree [5]<br>2. Agree [4]<br>3. Uncertain [3]<br>4. Disagree [2]<br>5. Strongly disagree [1]
32.	bmqs2	Having to give my child ARVs worries me	1. Strongly agree [5]<br>2. Agree [4]<br>3. Uncertain [3]<br>4. Disagree [2]<br>5. Strongly disagree [1]
33.	bmqs3	My child's life would be impossible without their ARVs	1. Strongly agree [5]<br>2. Agree [4]<br>3. Uncertain [3]<br>4. Disagree [2]<br>5. Strongly disagree [1]
34.	bmqs4	Without my child's ARVs he/she would be very ill	1. Strongly agree [5]<br>2. Agree [4]<br>3. Uncertain [3]<br>4. Disagree [2]<br>5. Strongly disagree [1]
35.	bmqs5	I sometimes worry about the long-term effects of my child's ARVs	1. Strongly agree [5]<br>2. Agree [4]<br>3. Uncertain [3]<br>4. Disagree [2]<br>5. Strongly disagree [1]
36.	bmqs6	My child's ARVs are a mystery to me	1. Strongly agree [5]<br>2. Agree [4]<br>3. Uncertain [3]<br>4. Disagree [2]<br>5. Strongly disagree [1]
37.	bmqs7	My child's health in the future will depend on their ARVs	1. Strongly agree [5]<br>2. Agree [4]<br>3. Uncertain [3]<br>4. Disagree [2]<br>5. Strongly disagree [1]
38.	bmqs8	My child's ARVs disrupt their life	1. Strongly agree [5]<br>2. Agree [4]<br>3. Uncertain [3]<br>4. Disagree [2]<br>5. Strongly disagree [1]
39.	bmqs9	I sometimes worry about my child becoming dependent on their ARVs	1. Strongly agree [5]<br>2. Agree [4]<br>3. Uncertain [3]<br>4. Disagree [2]<br>5. Strongly disagree [1]
40.	bmqs10	My child's ARVs protect them from becoming worse	1. Strongly agree [5]<br>2. Agree [4]<br>3. Uncertain [3]<br>4. Disagree [2]<br>5. Strongly disagree [1]
**Alcohol and drug use**			

People have different health habits. The following questions ask about your alcohol and drug use, past and current. An alcoholic beverage here refers to beer, wine, vodka, whiskey, local brew or a mixed drink.			
41. (a)	alc_use	How often have you had an alcoholic drink in the last 30 days?	1. Never [0]<br>2. Once a month [1]<br>3. 2 or 3 times a month [2]<br>4. Once or twice a week [3]<br>5. 3 or 4 times a week [4]<br>6. Nearly every day [5]<br>7. Daily [6]<br><br>If never, skip ahead to question 41
41 (b)	alc_qty	On days when you drank any alcoholic beverages in the last 30 days, how many drinks did you usually have altogether? By a drink we mean a can or glass of beer, a glass of wine, a shot of liquor, or a mixed drink with a shot of liquor?	1. 1 or 2 drinks per day [1]<br>2. 3 or 4 drinks per day [2]<br>3. 5 or 6 drinks per day [3]<br>4. 7 or 8 drinks per day [4]<br>5. 9 to 11 drinks per day [5]<br>6. 12 or more drinks per day [6]
41 (c)	alc_hvy	During the past 30 days, how often have you had 5 or more drinks of alcohol in a row, that is, within a couple of hours (for example 2 to 4 hours)?	1. Never [0]<br>2. Once a month [1]<br>3. 2 or 3 times a month [2]<br>4. Once or twice a week [3]<br>5. 3 or 4 times a week [4]<br>6. Nearly everyday [5]<br>7. Daily [6]
42. (a)	drg_use	Have you ever used marijuana or any other illicit drug?	1. Yes [1]<br>2. No (Skip the next question) [0]
42 (b)	drg_hvy	If you used it, have you used it within the past 6 months?	1. Yes [1]<br>2. No [0]
**Household food security**<br><br>Now I am going to ask you about the availability of food in your home.			
43. (a)	hfs1	In the past four weeks, did you worry that your household would not have enough food?	1. No (Skip the next question) [0]<br>2. Yes [1]
43 (b)	hfs2	How often did this happen?	1. Rarely (once or twice in the past four weeks) [1]<br>2. Sometimes (3 to 10 times in the past four weeks) [2]<br>3. Often (more than 10 times in the past four weeks) [3]
44. (a)	hfs3	In the past four weeks, were you or any household member not able to eat the kinds of food you preferred because of a lack of resources?	1. No (Skip next question) [0]<br>2. Yes [1]

44 (b)	hfs4	How often did this happen?	1. Rarely [1]<br>2. Sometimes [2]<br>3. Often [3]
45. (a)	hfs5	In the past four weeks, did you or any household member have to eat a limited variety of foods due to lack of resources?	1. No (Skip next question) [0]<br>2. Yes [1]
45 (b)	hfs6	How often did this occur?	1. Rarely [1]<br>2. Sometimes [2]<br>3. Often [3]
46. (a)	hfs7	In the past four weeks, did you or any household member have to eat some foods that you really did not want to eat because of lack of resources to obtain other types of food?	1. No (skip next question) [0]<br>2. Yes [1]
46 (b)	hfs8	How often did this happen?	1. Rarely [1]<br>2. Sometimes [2]<br>3. Often [3]
47. (a)	hfs9	In the past four weeks, did you or any household member have to eat a smaller meal than you felt you needed because there was not enough food?	1. No (skip next question) [0]<br>2. Yes [1]
47 (b)	hfs10	How often did this happen?	1. Rarely [1]<br>2. Sometimes [2]<br>3. Often [3]
48. (a)	hfs11	In the past four weeks, did you or any other household member have to eat fewer meals in a day because there was not enough food?	1. No (skip next question) [0]<br>2. Yes [1]
48 (b)	hfs12	How often did this happen?	1. Rarely [1]<br>2. Sometimes [2]<br>3. Often [3]
49. (a)	hfs13	In the past four weeks, was there ever no food to eat of any kind in your household<br>because of lack of resources to get food?	1. No (Skip next question) [0]<br>2. Yes [1]
49 (b)	hfs14	How often did this happen?	1. Rarely [1]<br>2. Sometimes [2]<br>3. Often [3]
50. (a)	hfs15	In the past four weeks, did you or any household member go to sleep at night hungry because there was not enough food.	1. No (skip next question) [0]<br>2. Yes [1]
50 (b)	hfs16	How often did this happen?	4. Rarely [1]<br>5. Sometimes [2]<br>6. Often [3]
51. (a)	hfs17	In the past four weeks, did you or any household member go a whole day	1. No (Skip next question) [0]<br>2. Yes [1]

		and night without eating anything because there was not enough food?	
51 (b)	hfs18	How often did this happen?	1. Rarely [1]<br>2. Sometimes [2]<br>3. Often [3]

SECTION C: ART Regimen Characteristics

Most people with children who have HIV have to strictly follow their regimen guidelines such as administering pills or syrups daily at specific times with fixed intervals between doses. We would like you to answer these questions to understand how people with children who have HIV are doing so as to improve your child and others' ART adherence.

No.	Code	Question	Response
**52.**	**ad_dose**	We would like you to show us HOW MUCH of the child's ARV medication they have taken DURING THE PAST 30 DAYS. We would be surprised if this was 100%.<br>Put a cross on the line below at the point showing your best guess about HOW MUCH of the medication the child has taken DURING THE PAST 30 DAYS. E.g. 0% means he/she has taken no medication; 50% means they have taken half of their medication, 100% means they have taken every single dose of medication.<br>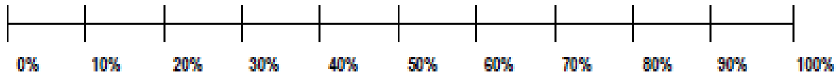 	Dose Adherence level:<br>……… %
**53.**	**ad_time**	The following question is about the TIMING OF THE DOSES of the child's ARV drugs. We would like to know HOW MANY DOSES of the medication you have administered WITHIN 2 HOURS OF THE CORRECT TIME DURING THE PAST 30 DAYS. That is how many did you give 2 hours or less away from the correct time. We would be surprised if this was 100% for most people too.<br>What do we mean by the 'correct time'? Well, if you are supposed to give the child's medication twice per day, the 'correct time' to give that Medication is every 12 hours exactly.<br>As explained above, put a cross on the line below at the point showing your best guess about HOW MUCH of the child's medication you gave WITHIN 2 HOURS OF THE CORRECT TIME DURING THE PAST 30 DAYS.<br>E.g. 0% means you haven't given any dose of the Medication within 2 hours of the correct time, 50% means you have given half of the prescribed doses within 2 hours of the correct time and 100% means you have given every single dose of the Medication within 2 hours of the correct time.<br>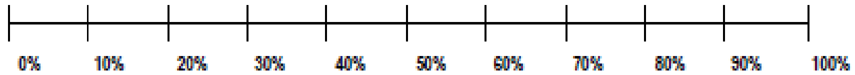 	Timing Adherence level:<br>………(%)
	**Reasons for missing a dose:**<br>People may miss taking their medications for various reasons. I'm going to ask you about the reasons why you may have missed giving your child their ARV medications.		

**54.**	**reason**	Please indicate which of these has been the main reason you have missed giving your child their ARVs:	1. I haven't missed giving my child medicine [1]<br>2. Were away from home [2]<br>3. Were busy with other things [3]<br>4. Simply forgot [4]<br>5. Had too many pills to administer [5]<br>6. Wanted to avoid side effects [6]<br>7. Did not want others to notice you giving the child medication [7]<br>8. I had a change in daily routine [8]<br>9. Felt like the drug was toxic/harmful [9]<br>10. I slept through dose time [10]<br>11. The child slept through the dose time [11]<br>12. The child felt sick or ill [12]<br>13. I felt sick or ill [13]<br>14. I felt depressed or overwhelmed [14]<br>15. I had problems administering the pills/syrups at specified times (e.g. with meals, on empty stomach, etc.) [15]<br>16. We ran out of pills [16]<br>17. The child felt good and I didn't feel the pills were necessary [17]<br>18. Other (specify) [18] reason2

SECTION D: Health system factors

Now I am going to ask you about the child's healthcare providers and services at this facility

**No.**	**Code**	**Question**	**Response**
**Relationship with health care providers (HCPs)**<br>Each item below is a statement about your relationship with the child's HCP. This is the nurse or doctor that attended to your child today. Please click one response that indicates how you would agree with each sentence.			
55.	rshp1	Your child's healthcare provider (HCP) greeted you and your child in a way that made you feel comfortable.	1. Strongly disagree [1]<br>2. Somewhat disagree [2]<br>3. Neither agree nor disagree [3]<br>4. Somewhat agree [4]<br>5. Strongly agree [5]
56.	rshp2	The HCP discussed your reason(s) for coming today?	1. Strongly disagree [1]<br>2. Somewhat disagree [2]<br>3. Neither agree nor disagree [3]

			4. Somewhat agree [4]<br>5. Strongly agree [5]
57.	rshp3	The HCP encouraged you to express your thoughts concerning your child's health problems.	1. Strongly disagree [1]<br>2. Somewhat disagree [2]<br>3. Neither agree nor disagree [3]<br>4. Somewhat agree [4]<br>5. Strongly agree [5]
58.	rshp4	The HCP listened carefully to what you had to say.	1. Strongly disagree [1]<br>2. Somewhat disagree [2]<br>3. Neither agree nor disagree [3]<br>4. Somewhat agree [4]<br>5. Strongly agree [5]
59.	rshp5	The HCP understood what you had to say.	1. Strongly disagree [1]<br>2. Somewhat disagree [2]<br>3. Neither agree nor disagree [3]<br>4. Somewhat agree [4]<br>5. Strongly agree [5]
60.	rshp6	The HCP gave you as much information as you wanted.	6. Strongly disagree [1]<br>7. Somewhat disagree [2]<br>8. Neither agree nor disagree [3]<br>9. Somewhat agree [4]<br>10. Strongly agree [5]
61.	rshp7	The HCP checked to see if the treatment plan was acceptable to you and your child.	1. Strongly disagree [1]<br>2. Somewhat disagree [2]<br>3. Neither agree nor disagree [3]<br>4. Somewhat agree [4]<br>5. Strongly agree [5]
62.	rshp8	The HCP encouraged you to ask questions	1. Strongly disagree [1]<br>2. Somewhat disagree [2]<br>3. Neither agree nor disagree [3]<br>4. Somewhat agree [4]<br>5. Strongly agree [5]
63.	rshp9	The HCP responded to your questions and concerns.	1. Strongly disagree [1]<br>2. Somewhat disagree [2]<br>3. Neither agree nor disagree [3]<br>4. Somewhat agree [4]<br>5. Strongly agree [5]
64.	rshp10	The HCP involved you in decisions about your child's health as much as you wanted.	1. Strongly disagree [1]<br>2. Somewhat disagree [2]<br>3. Neither agree nor disagree [3]<br>4. Somewhat agree [4]<br>5. Strongly agree [5]
65.	rshp11	The HCP checked to be sure you understood everything.	1. Strongly disagree [1]<br>2. Somewhat disagree [2]<br>3. Neither agree nor disagree [3]<br>4. Somewhat agree [4]<br>5. Strongly agree [5]
66.	rshp12	The HCP spent the right amount of time with your child.	1. Strongly disagree [1]<br>2. Somewhat disagree [2]<br>3. Neither agree nor disagree [3]<br>4. Somewhat agree [4]<br>5. Strongly agree [5]

67.	rshp13	Overall, you were satisfied with your child's visit to the doctor today.	1. Strongly disagree [1]<br>2. Somewhat disagree [2]<br>3. Neither agree nor disagree [3]<br>4. Somewhat agree [4]<br>5. Strongly agree [5]									
	**Satisfaction with the quality of service received at the health facility**<br><br>Please indicate your satisfaction with each of the following aspects of this clinic by selecting a number from 1 to 10, where 1 means not satisfied and 10 means extremely satisfied.											
68.	sat1	Physical environment	1	2	3	4	5	6	7	8	9	10
69.	sat2	Equipment and facilities	1	2	3	4	5	6	7	8	9	10
70.	sat3	Appointment arrangement	1	2	3	4	5	6	7	8	9	10
71.	sat4	Waiting time	1	2	3	4	5	6	7	8	9	10
72.	sat5	Service of the pharmacy	1	2	3	4	5	6	7	8	9	10
73.	sat6	Support staff	1	2	3	4	5	6	7	8	9	10
74.	sat7	Your case physician's professionalism	1	2	3	4	5	6	7	8	9	10
75.	sat8	Explanations given by your case physician	1	2	3	4	5	6	7	8	9	10
76.	sat9	Consultation time	1	2	3	4	5	6	7	8	9	10

We have come to the end of this survey. We thank you for your time. We greatly appreciate your participation in this study.
