# Supplementary material for: Multi-measure assessment of adherence to antiretroviral therapy among children under five years living with HIV in Jinja, Uganda
Source: BMC Public Health. 2020 Aug 31;20:1319. doi: 10.1186/s12889-020-09430-w (PMC7457490; doi:10.1186/s12889-020-09430-w)
Supplement: Supplementary file 2 — Additional file 2. Data abstraction form: Data abstraction form used for extraction of the children’s medical records. [file 12889_2020_9430_MOESM2_ESM.pdf]

DATA ABSTRACTION FORM

Factors associated with non-adherence to anti-retroviral therapy among children under five years in Jinja district.

Date of data abstraction:<br>(dd/mm/yyyy)	Interviewer number:
Start time: (am/pm)	[part_id] Participant ID:
End time: (am/pm)	[facility] Health facility:

<table>
<tr><th>No.</th><th>Code</th><th>Item</th><th colspan="4">Options</th></tr>
<tr><td></td><td colspan="6">Medical Record Review
For the following questions, review the patient's medical records.</td></tr>
<tr><td>1.</td><td>dob_cd</td><td>Child's date of birth</td><td colspan="4">(dd/mm/yyyy)</td></tr>
<tr><td>2.</td><td>sex_rec</td><td>Sex</td><td colspan="4">1. Female [1]
2. Male [0]</td></tr>
<tr><td>3.</td><td>vrl1</td><td>Record the child's baseline HIV viral load measurement at ART initiation.</td><td colspan="4">……………………..</td></tr>
<tr><td>4.</td><td>vrl2</td><td>Record the child's most recent HIV viral load measurement.</td><td colspan="4">………………………</td></tr>
<tr><td></td><td colspan="6">Medication Record Review
For the next questions, review the patient's HIV Care/ART card</td></tr>
<tr><td>5.</td><td>reg_curr</td><td>ART regimen the child is currently on</td><td colspan="4">1. ABC+3TC+LPV/r [1]
2. ABC+3TC+EFV [2]
3. ABC+3TC+NVP [3]
4. AZT+3TC+LPV/r [4]
5. AZT+3TC+NVP [5]
6. AZT+3TC+EFV [6]
7. Other (Specify) [7] reg_curr2_______</td></tr>
<tr><td rowspan="2">6.</td><td rowspan="2">form</td><td rowspan="2">Formulation of the ARV drugs.</td><td>Drug 1 [form1]</td><td>Drug 2 [form2]</td><td>Drug 3 [form3]</td><td>Drug 4 [form4]</td></tr>
<tr><td>1.Solid
2.Liquid</td><td>1.Solid
2.Liquid</td><td>1.Solid
2.Liquid</td><td>1.Solid [1]
2.Liquid [2]</td></tr>
<tr><td rowspan="2">7.</td><td rowspan="2">freq</td><td rowspan="2">Dosing frequency</td><td>Drug 1 [freq1]</td><td>Drug 2 [freq2]</td><td>Drug 3 [freq3]</td><td>Drug 4 [freq4]</td></tr>
<tr><td></td><td></td><td></td><td></td></tr>
<tr><td>8.</td><td>date_stat</td><td>Date of first ARV drug pick-up (date started on ART)</td><td colspan="4">(dd/mm/yyyy)</td></tr>
<tr><td>9.</td><td>stage</td><td>What was the patient's clinical stage on initiation to ART?</td><td colspan="4">………………….</td></tr>
</table>

10.	reg_sta t	ART regimen picked up on first pick-up/initiation of ART.	1. ABC+3TC+LPV/r [1]<br>2. ABC+3TC+EFV [2]<br>3. ABC+3TC+NVP [3]<br>4. AZT+3TC+LPV/r [4]<br>5. AZT/3TC/NVP [5]<br>6. AZT/3TC/EFV [6]<br>7. Other (Specify) [7] reg_stat2_________
11.	sidef_h f	Have side effects/toxicity been reported on the child's ART card?	1. Yes [1]<br>2. No [0]
12.	date_fi rst	For this question, review the patient's prescription and dispensing records for the past 90 days. Identify the date of the first supply of medicine in the last 90 days.	__________________ (dd/mm/yyyy)
13.	days_u nc	How many days from the date of first supply till today was the patient not covered by the ARV drug supply for all drugs in the ART regimen? *(consider all days when the patient did not have supply of any or all drugs in the regimen)*	_________ (No. of days not covered)
14.	stock_ out	Was any of the drugs in the child's regimen out of stock at any time in the past 3 months?	1. No [0]<br>2. Yes [1]
15.	no_refi ll	How many refills has the child had in the past 3 months?	__________________ (number)
16.	ad_rec	What is the adherence level recorded in the patient's file?	1. G (Good: ≥ 95%) [3]<br>2. F (Fair: 85 – 94%) [2]<br>3. P (Poor: < 85%) [1]
